# The reverse buffering effect of workplace attachment style on the relationship between workplace bullying and work engagement

**DOI:** 10.3389/fpsyg.2023.1112864

**Published:** 2023-02-23

**Authors:** Jean-Félix Hamel, Pierpaolo Iodice, Klara Radic, Fabrizio Scrima

**Affiliations:** ^1^Centre de Recherche sur les Fonctionnements et les Dysfonctionnements Psychologiques (CRFDP) (UR7475), University of Rouen Normandy, Rouen, France; ^2^Centre d’Études des Transformations des Activités Physiques et Sportives (CETAPS) (UR3832), University of Rouen Normandy, Rouen, France; ^3^Paris Nanterre University, Nanterre, France

**Keywords:** aggression, place attachment, workplace attachment, work engagement, job demands–resources model, bullying

## Abstract

Using the Job Demands-Resources model, this study investigates workplace attachment styles as predictors of work engagement and moderators of the well-established disengaging effect of workplace bullying. As a personal resource, we hypothesized that secure workplace attachment would foster work engagement, whereas both types of insecure workplace attachment (i.e., avoidant and preoccupied) would do the opposite. Previous work also led us to expect the relationship between workplace bullying and engagement to be stronger when targets expect it to act as job resource (i.e., secure workplace attachment) and weaker when their working model is consistent with workplace aggression–i.e., reverse buffering effects. Using the PROCESS macro, we tested these hypotheses in a convenience sample of French office employees (*N* = 472) who completed an online survey. Secure workplace attachment was associated with higher work engagement while insecure workplace attachment and bullying perceptions related negatively with work engagement. Supporting our hypotheses, feeling exposed to workplace bullying was most associated with disengagement in employees with a secure workplace attachment style and less so in others. Far from recommending insecure bonds as protection, our results rather highlight the need to prevent all forms of workplace aggression, thereby allowing employees to rely on their work environment as a job resource.

## 1. Introduction

A vast corpus of research has established why workplace bullying warrants attention from work and organizational scholars. This growing literature has supported significant efforts to legislate and regulate it worldwide (Cobb, 2017). Despite studies approaching the subject from multiple theoretical frameworks and using different measurement methods, it is possible to state that this phenomenon has a global prevalence (Nielsen et al., 2011; León-Pérez et al., 2021). This pervasive social issue has been shown to be overwhelmingly detrimental to targets, organizations and society at large (e.g., Samnani and Singh, 2012; Conway et al., 2018; Rai and Agarwal, 2018).

Given its far-reaching scope, past research first investigated the predictors and outcomes of workplace bullying. However, the need to integrate these findings with established theories has since been recognized as critical to the advancement of the field (Nielsen and Einarsen, 2018). In line with this recommendation, previous work relied on the self-determination theory to explain the relationship between workplace bullying and work engagement (Goodboy et al., 2020). Very few studies (e.g., McGregor et al., 2016), however, framed this relationship using the Job Demands-Resources model (JD-R, Demerouti et al., 2001).

In recent years, research also started clarifying how and when workplace bullying relates to other variables (i.e., mediators and moderators). Notably, a number of individual factors have been associated with reverse buffering effects on the relationship between exposure to workplace bullying and its outcomes (e.g., Britton et al., 2012; Nielsen et al., 2022). Regarding work engagement (Schaufeli et al., 2002), several preventive factors related to how employees experience their psychosocial work environment have been uncovered, such as work climate (Einarsen et al., 2018) or power imbalance perceptions (Nielsen et al., 2022). Since they imply distinct sets of expectations regarding workplace aggression, we propose *workplace attachment styles*–i.e., the type of affective relationship employees develop with their physical work environment (Scrima et al., 2017)–may play a similar role.

While a number of recent works have investigated the relationship between workplace bullying, work engagement and its moderators (e.g., Einarsen et al., 2018; Tagoe and Amponsah-Tawiah, 2020), studies have yet to investigate the role of workplace attachment styles (i.e., secure, avoidant, and preoccupied) in shaping both work engagement and the influence of violent behaviors such as bullying. The present research first investigates the relationship between workplace bullying and work engagement through the lens of the JD-R model. Second, we build on this discussion by hypothesizing as to the influence of workplace attachment styles on work engagement. Finally, we investigate the reverse buffering effect of workplace attachment styles on the workplace bullying–work engagement relationship.

## 2. Theoretical framework

### 2.1. Workplace bullying and the job demands-resources model

The study of workplace bullying has seen tremendous growth since it first appeared in a scientific journal about 30 years ago (Leymann, 1990). In the European tradition, workplace bullying designates situations in which an employee is frequently and persistently subjected to willful negative behaviors by one or more colleagues (whether subordinates, peers or supervisors) (Einarsen et al., 2011). The systematic exposition of employees to such behaviors tends to render targets powerless to defend themselves and submissive to further abuse by perpetrators. Workplace bullying appears specific in the high frequency, intensity, and persistency of the underlying mistreatments (Nielsen and Einarsen, 2018)–thus, it is widely considered as one of the most detrimental job stressors (e.g., Trépanier et al., 2015).

Originating from the study of burnout (Demerouti et al., 2001), the job demands-resources (JD-R) model has since been recognized as an overarching theory of job stress factors that may be applied to a wide variety of occupations and conditions (Bakker et al., 2023). This versatility stems from its base theoretical propositions. First, job characteristics can be categorized into two broad groups either referring to aspects of a job that (1) require sustained efforts and are thus associated with costs (i.e., *job demands*) or (2) “help to either achieve work goals, reduce job demands […] or stimulate personal growth, learning and development” (i.e., *job resources*, Bakker et al., 2014, p. 392). Second, job demands are theorized to instigate a health impairment process *via* strain, whereas job resources are thought to foster a motivational process through the satisfaction of basic needs. Finally, these *dual processes* (and the interactions between them) provide the basis for demands and resources influencing organizational outcomes (Bakker and Demerouti, 2007).

Depending on their purpose, past studies exploring workplace bullying within the confines of the JD-R model described it as either a job demand (e.g., McGregor et al., 2016) or an outcome associated with high-demands and low-resources work environments (e.g., Van den Broeck et al., 2011; Nel and Coetzee, 2020). The second perspective aligns with the underlying theory stating that job demands (e.g., interacting with colleagues or supervisors) are not inherently negative, but can indeed turn into job stressors given the right conditions (Bakker and Demerouti, 2007). Specifically, prior research suggests *hindrance stressors*–exclusively detrimental demands or conditions which prevent or interfere with goal achievement (Van den Broeck et al., 2010)–are negatively associated with work engagement and positively so with exhaustion.

These observations are consistent with several meta-analyses (e.g., Nielsen and Einarsen, 2012), systematic reviews (e.g., Nielsen et al., 2017b) or longitudinal studies (e.g., Rodríguez-Muñoz et al., 2009; Trépanier et al., 2015) outside the scope of the JD-R model. As it greatly hinders employee autonomy, it is no surprise previous work observed the negative effect of workplace bullying on work engagement to be mediated by the thwarting of fundamental needs and the ensuing lack of intrinsic motivations to work (Goodboy et al., 2020)–which ties in to the motivational process of the JD-R model. Overall, this literature establishes that employees who feel subjected to bullying in the workplace are expected to disengage from their work.

*H_1_: Workplace bullying is negatively related with work engagement.*

### 2.2. Workplace attachment styles

#### 2.2.1. The influence of workplace attachment styles on work engagement

Originating from the influential work of Bowlby (1969), attachment theory can be considered one of the most prominent frameworks used by researchers to discuss human interactions to date (Cassidy and Shaver, 2018). It supposes the innate existence of an *attachment behavioral system*, which actively encourages individuals to look for the support of others in times of need. Depending on the quality and consistency of this support, individuals form distinct working models of relationships–i.e., *attachment styles*–which may influence many social and organizational phenomena (Bartholomew and Horowitz, 1991; Yip et al., 2018). A staple of environmental psychology for the past 60 years (Fried and Gleisher, 1961; Lewicka, 2011), the study of the emotional connection between people and places (i.e., place attachment) has recently been integrated into this literature. Individuals tend to develop specific patterns of attachment to their place of work which are comparable to adult attachment styles (i.e., secure, avoidant and preoccupied) and conveyed by corresponding sets of observable behaviors in the workplace (Scrima et al., 2017).

A *secure* workplace attachment style is defined by a positive view of both the self and the workplace; these individuals tend to perceive their work environment as a *safe space* wherein their worth is acknowledged and the satisfaction of their needs facilitated, and thus seek its proximity. Conversely, employees with an *avoidant* attachment style carry negative expectations of their workplace (i.e., considering it a threat rather than a resource) and maintain a positive self-image; counter-dependent, these individuals no longer seek its proximity or support, actively attempting to handle detrimental situations alone (Mikulincer et al., 2003). With a positive view of the workplace and a negative self-representation, *preoccupied* employees feel unworthy and anticipate rejection; anxious to remain close to their object of attachment, they tend to invest it all the more, which may come at the cost of a more strenuous work experience (Leiter et al., 2015). Employees with insecure workplace attachment styles (i.e., avoidant and preoccupied) have been shown to be more exhausted than others (Scrima et al., 2021). Secure workplace attachment was also found to promote organizational citizenship behaviors in health workers (Nonnis et al., 2022).

Previous work using the JD-R model has clearly established the role of individual characteristics in predicting work engagement. Indeed, several meta-analyses (e.g., Mäkikangas et al., 2013; Mazzeti et al., 2021) suggest factors such as optimism, self-efficacy, or proactive personality are not only consistent determinants of work engagement but also more influential than either social or job resources. In their recent review, Bakker et al. (2023) define these *personal resources* as “positive self-evaluations that refer to individuals’ sense of their ability to control and impact their environment successfully” (p. 33). Workplace attachment styles imply different self-evaluations, representations of the workplace and associated tendencies to seek or avoid its proximity (Scrima et al., 2017). Thus, we consider *secure* workplace attachment a personal resource which may positively influence the extent to which employees mobilize their physical work environment–a quintessential job resource–to further engage with their work; as it implies either a negative self-representation and anxiety (i.e., preoccupied) or considering the environment as a threat despite a positive self-evaluation (i.e., avoidant), *insecure* workplace attachment should hinder work engagement.

*H_2_: Secure workplace attachment is positively related with work engagement.*

*H_3_: Avoidant workplace attachment is negatively related to work engagement.*

*H_4_: Preoccupied workplace attachment is negatively related to work engagement.*

#### 2.2.2. Workplace attachment style as a moderator

Another proposition of the JD-R model is that individual characteristics may moderate the impact of job stressors on employee wellbeing (Bakker et al., 2023). Past theoretical claims (Kahn and Byosserie, 1992, cited by Bakker and Demerouti, 2007, p. 314) and empirical findings (e.g., Bakker and Sanz-Vergel, 2013) suggest these factors may shape employees’ ability to handle stressors in different ways, including changing the perceptions and cognitions associated with the work environment. As such, workplace attachment styles should provide individuals with distinct frameworks for perceiving and interpreting violence in the workplace, thus influencing its consequences on work engagement. This would be consistent with a key tenet of attachment theory: in addition to their working model of relationships, individuals also internalize corresponding ways to regulate threats (Yip et al., 2018). For example, as secure individuals are generally optimistic and confident that others will help when difficulties arise (Mikulincer and Shaver, 2015), they should react to aggression differently than those who expect to be mistreated in the first place.

Prior work suggests a number of individual factors determine if and how the same objective work situation influences work engagement (Mäkikangas et al., 2013). Using the JD-R model, Li and Mao (2014) observed individuals with a proactive personality were more engaged than others when receiving social support. In another study, avoidant attachment interacted with autonomy in predicting work engagement (Littman-Ovadia et al., 2013); since workplace bullying tends to hinder employee autonomy, there is reason to think it may also interact with attachment styles. Moreover, recent work suggests workplace bullying can indeed involve the workplace (Ein-Eli and Rioux, 2022); for example, deliberately placing work space in isolated locations, intentionally destroying, stealing or sabotaging work materials are typical behaviors which use the physical-spatial characteristics of the workplace to bully employees (Fox and Cowan, 2015). This leads us to believe workplace bullying may also interact with *workplace* attachment styles in shaping employees’ work engagement.

**FIGURE 1:**
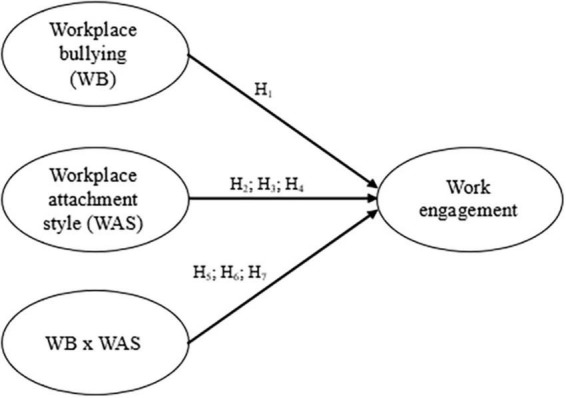
Theoretical model.

Over the past decade, numerous studies exploring the influence of individual factors on the outcomes of workplace bullying observed *reverse buffering* effects. In other words, variables usually considered as preventive factors such as optimism (Britton et al., 2012), the ability to defend oneself (Nielsen et al., 2017a), or power balance between target and perpetrator (Nielsen et al., 2022) actually enhanced the effects of workplace bullying. We propose a similar pattern should be observed in this study: the more positive expectations individuals have anchored on their work environment, the more instances of workplace bullying taking place in this environment should disengage them from their work. Hence, secure individuals should disengage more intensely when exposed to this sort of aggression. Conversely, these violent behaviors should not contrast as much with the expectations of insecure individuals–whether they see their work environment as a threat (i.e., avoidant) or themselves as unworthy of any other kind of treatment (i.e., preoccupied). An overview of the theoretical model has been provided (Figure 1).

*H_5_: Individuals with a secure workplace attachment style experience a stronger disengagement from their work due to workplace bullying compared to other individuals.*

*H_6_: Individuals with an avoidant workplace attachment style experience a weaker disengagement from their work due to workplace bullying compared to other individuals.*

*H_7_: Individuals with a preoccupied workplace attachment style experience a weaker disengagement from their work due to workplace bullying compared to other individuals.*

## 3. Materials and methods

### 3.1. Sample and procedure

A convenience sample of 500 employees was recruited online through posts on various professional (e.g., LinkedIn) and non-professional (e.g., Facebook) social media platforms. The study was carried out following the American Psychological Association (2017) ethical principles guidelines. Before being asked to fill our survey, participants were made aware of the two retained inclusion criteria: (1) being an *office employee* (i.e., administrative work, desk-job) and (2) having been working in the same organization for at least 1 year. If so, participants were then informed about the aims of the study–that is, to study the relationship between workplace bullying and work engagement. They were all asked to confirm their informed consent to participate and reminded that they could abandon the survey at any time. The survey was completely anonymous and involved no monetary compensation.

After eliminating 28 participants, as they did not fully respond to the survey, our sample was comprised of 472 office workers, 31.1% men and 68.9% women, aged between 20 and 65 (*M* = 41.05, *SD* = 11.94) and with a length of service between 1 and 42 years (*M* = 16.02, *SD* = 11.39). Of these, 89.4% reported working in the public sector. We conducted an *a posteriori* power analysis to test the power our sample *via* G*Power 3.1 software (Faul et al., 2007). Assuming conservative criteria of an average effect size (*f*^2^ = 0.15) and an acceptable probability of error (α = 0.05), we obtained a β of 0.95.

### 3.2. Measures

The first section of the survey was dedicated to socio-demographic variables such as age, sex, length of service, and professional sector. The objective of the three subsequent sections was to measure the variables contained in the hypothesized moderation models. Workplace bullying was evaluated using the Short Negative Acts Questionnaire (SNAQ, Notelaers et al., 2019). This tool is composed of nine items (e.g., Being ignored or excluded) with answers situated on a 5-point Likert scale from 1 (*never*) to 5 (*always*). Participants had to indicate how often, in the past 12 months, they found themselves in one of the situations described by the items. In our study, confirmatory factor analysis of the mono-factorial structure provided the following fit indices (χ^2^/*df* = 1.83, *CFI* = 0.99, *NNFI* = 0.98, *SRMR* = 0.03) suggesting an excellent adequacy of the model to the collected data. The scale had excellent internal consistency (ω = 0.86).

Workplace attachment style was measured using the Workplace Attachment Style Questionnaire (WASQ, Scrima, 2020). This scale consists of 15 items (five per workplace attachment style). Examples include “I prefer not to go to certain places in my organization” (avoidant), “I enjoy the time that I spend in my workplace” (secure) and “I find it difficult to feel at ease at my workplace” (preoccupied). The scale provides a 7-point Likert response mode from 1 (*totally disagree*) to 7 (*totally agree*). In the present study, the confirmatory factor analysis indicates, as predicted by the original structure (Scrima, 2020), a correlated three-factor structure (*χ^2^/df* = 2.81, *CFI* = 0.96, *NNFI* = 0.95, *SRMR* = 0.06). Since both insecure dimensions (i.e., avoidant and preoccupied) tend to be highly correlated, we also tested a correlated two-factor structure (*χ^2^/df* = 3.68, *CFI* = 0.94, *NNFI* = 0.94, *SRMR* = 0.07). The three-factor model showed significantly better fit to our data (Δ*χ^2^* = 79, Δ*df* = 2, *p* < 0.001). Thus, insecure workplace attachment styles were considered as distinct in further analyses. McDonald’s ω was satisfactory for each subscale (0.81, 0.82, and 0.82, respectively).

Work engagement was assessed with the Utrecht Work Engagement Scale–9 (UWES-9, Balducci et al., 2010). This tool composed of 9 items–three per dimension of the underlying theoretical model: vigor (e.g., At my work, I feel bursting with energy), dedication (e.g., I am enthusiastic about my job), and absorption: (e.g., I feel happy when I am working intensely). Responses are given on a 7-point Likert scale varying from 1 (*never*) to 7 (*always*). In the present study, we chose to use the overall work engagement score as suggested by De Bruin and Henn (2013). A measurement model with three latent factors plus a second order factor shows satisfactory fit indices (*χ^2^/df* = 2.76, *CFI* = 0.99, *NNFI* = 0.97, *SRMR* = 0.02). The scale proved satisfactory regarding internal consistency (ω = 0.91).

## 4. Results

### 4.1. Preliminary analysis

Because cross-sectional designs could be affected by common method bias (Podsakoff et al., 2012), we compared five models using AMOS 4.0 software (Arbuckle and Wothke, 1999). In the first model, all items of the five variables saturated on a single latent factor. The second model contained two covaried latent factors, the first of which encompassed all items of the workplace attachment styles questionnaire while the second latent factor accounted for items from the workplace bullying and work engagement scales. The third model had three covaried latent factors, one for each construct (i.e., workplace attachment style, workplace bullying, and work engagement). In the fourth model, the workplace attachment factor was split with items representing secure and insecure workplace attachment (i.e., avoidant and preoccupied) loading on distinct and covaried latent factors. In the fifth model, we covaried five latent factors: three workplace attachment styles, workplace bullying, and work engagement.

The results (Table 1) indicate that the only model showing satisfactory fit indices is model 5. In addition, the Δχ^2^ test suggests a substantial improvement (*p* < 0.001) over the model with four latent factors. Finally, we tested a sixth model wherein a common method variance latent factor loading on all items was added to model 5. Previous work suggests these models should be compared using CFI rather than chi-square [see Jiang et al. (2019)]. As the difference in CFI between model 5 and the model containing a common method variance factor was negligible (Δ ≤ 0.01), these models can be considered as functionally equivalent. These results highlight the absence of common method bias.

**TABLE 1:** Measurement model.

Model	*χ^2^*	df	*p*	χ^2^/df	CFI	NNFI	SRMR	Δ χ^2^	Δ df	*p*
One factor	4,095	484	<0.001	8.46	0.61	0.58	0.13			
Correlated 2-factor	3,062	480	<0.001	6.38	0.72	0.69	0.13	1,033	4	<0.001
Correlated 3-factor	2,022	477	<0.001	4.24	0.83	0.82	0.09	1,040	3	<0.001
Correlated 4-factor	1,427	474	<0.001	3.01	0.90	0.89	0.08	595	3	<0.001
Correlated 5-factor	1,339	470	<0.001	2.85	0.91	0.90	0.07	88	4	<0.001

### 4.2. Descriptive statistics

Table 2 shows the correlation indices between the variables under study. Sex is negatively associated with workplace bullying (*p* < 0.05), suggesting that women score higher than their male colleagues. No significant correlation is observed between age and the other relevant variables under study. Workplace bullying appears to be negatively correlated (*p* < 0.01) with work engagement. Employees who experience workplace bullying tend to also feel less engaged at work. The three workplace attachment styles are found to be correlated with one another (*p* < 0.01). Specifically, secure workplace attachment shows negative correlations with the two insecure workplace attachment styles, which, conversely, are positively correlated with each other. Furthermore, secure workplace attachment style is negatively correlated with workplace bullying (*p* < 0.01) and positively correlated with work engagement (*p* < 0.01), while the two insecure workplace attachment styles are positively correlated with workplace bullying (*p* < 0.01) and negatively correlated with work engagement (*p* < 0.01). Employees with an insecure workplace attachment style tend to feel more frequently exposed to workplace bullying, whereas their secure counterparts report less such perceptions.

**TABLE 2:** Means, standard deviations, and zero-order correlations (ω on the diagonal).

		Min–Max	Mean	SD	1	2	3	4	5	6	7
1	Age	20–65	41.05	11.94	–						
2	Sex	–	–	–	0.12*	–					
3	Workplace bullying	1–5	1.95	0.73	0.09	−0.09*	(0.86)				
4	Avoidant WA	1–7	2.45	1.22	−0.01	0.07	0.44**	(0.81)			
5	Secure WA	1–7	4.11	1.15	−0.03	0.02	−0.27**	−0.38**	(0.82)		
6	Preoccupied WA	1–7	2.25	1.25	−0.06	0.08	0.44**	0.71**	−0.36**	(0.82)	
7	Work engagement	1–7	4.99	0.98	0.07	0.07	−0.36**	−0.35**	0.41**	−0.26**	(0.91)

*N* = 472. WA, workplace attachment, *p < 0.05, **p < 0.01. McDonald’s ω are in the diagonal.

### 4.3. Hypothesis testing

To test our hypotheses, we used the PROCESS macro for SPSS (Hayes, 2017). More specifically, we tested three moderation models (one for each workplace attachment style) using PROCESS model 1. To assess the significance of the effects, the lower (LL) and upper (UL) levels of the 95% confidence interval obtained from 5,000 bootstrap samples were used. Sex and age were included as covariates in all models. Regarding our first hypothesis, namely that workplace bullying is negatively associated with work engagement, results indicate that this effect is significant in all three models [secure (LL = −0.37, UL = −0.20), avoidant (LL = −0.39, UL = −0.20), and preoccupied (LL = −0.46, UL = −0.26)]. Secure workplace attachment (Table 3) has a significant positive direct effect (LL = 0.27, UL = 0.43) on work engagement, yet does not significantly moderate the effect between workplace bullying and work engagement (LL = −0.1, UL = 0.01). These findings do not corroborate our fifth hypothesis. However, the conditional effects table (Table 4) indicates a protective effect of low levels of secure attachment on the relationship between workplace bullying and work engagement.

**TABLE 3:** Moderation analysis of secure workplace attachment.

	Work engagement			
			**95% CI**	
	**β**	**SE**	**LL**	**UL**
Workplace bullying	−0.29	0.04	−0.37	−0.20
Secure workplace attachment	0.35	0.04	0.27	0.43
Interaction	−0.05	0.03	−0.11	0.01
Covariates				
Age	0.10	0.04	0.02	0.18
Sex	0.03	0.04	−0.05	0.11
	*R*^2^ = 25%			
	*F*_(5,466)_ = 23.98, *p* < 0.001			

**TABLE 4:** Conditional effect of focal predictor.

			95% CI	
**Secure workplace attachment**	**Effect**	**SE**	**LL**	**UL**
−1SD	−0.24	0.05	−0.34	−0.15
Mean	−0.29	0.04	−0.37	−0.20
+1SD	−0.33	0.06	−0.45	−0.22

**FIGURE 2:**
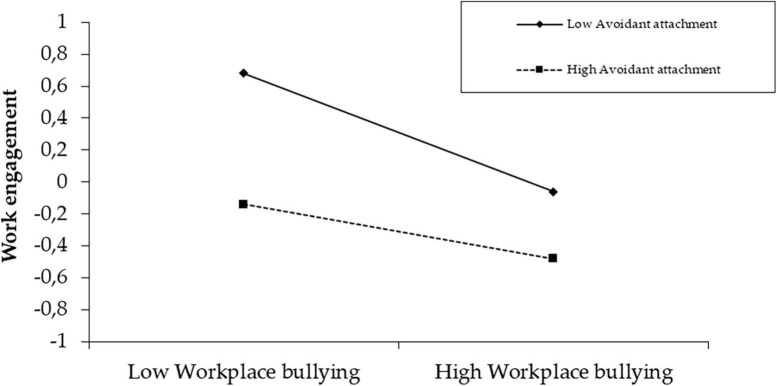
Simple slope analysis of the moderating effect of avoidant workplace attachment.

The second model investigates the moderating effect of avoidant workplace attachment style. The results indicate that avoidant workplace attachment style is negatively associated with work engagement (LL = −0.37, UL = −0.19) and moderates the relationship between workplace bullying and work engagement, explaining 20% of the variance of work engagement (Table 5). In addition, conditional effects analysis (Table 6) and simple slope analysis (Figure 2) indicate that the moderating effect of avoidant workplace attachment style is significant for low (*t* = −6.15, *p* < 0.001), mean (*t* = −6.01, *p* < 0.001), and high (*t* = −3.89, *p* < 0.001) avoidant attachment scores. That is, the lower the level of avoidant workplace attachment, the stronger the negative impact of workplace bullying on work engagement. Hypotheses 3 and 6 are supported by these results.

**TABLE 5:** Moderation analysis of avoidant workplace attachment.

	Work engagement			
			**95% CI**	
	**β**	**SE**	**LL**	**UL**
Workplace bullying	−0.29	0.05	−0.39	−0.20
Avoidant workplace attachment	−0.28	0.05	−0.37	−0.19
Interaction	0.10	0.03	0.04	0.16
Covariates				
Age	0.08	0.04	−0.00	0.16
Sex	0.05	0.04	−0.03	0.13
	*R*^2^ = 20%			
	*F*_(5,466)_ = 11.18, *p* < 0.001			

**TABLE 6:** Conditional effect of focal predictor.

			95% CI	
**Avoidant workplace attachment**	**Effect**	**SE**	**LL**	**UL**
−1SD	−0.39	0.06	−0.52	−0.27
Mean	−0.29	0.05	−0.39	−0.20
+1SD	−0.19	0.05	−0.29	−0.10

**FIGURE 3:**
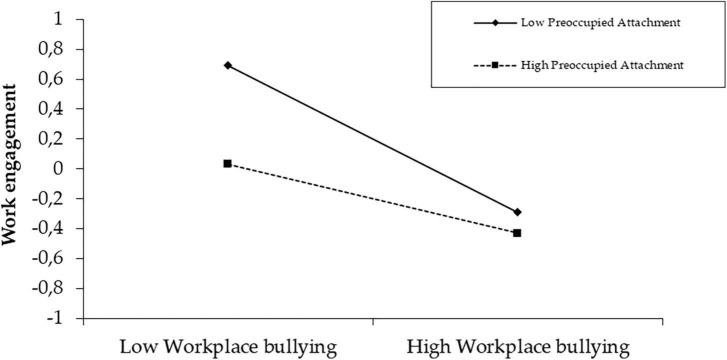
Simple slope analysis of the moderating effect of preoccupied workplace attachment.

Finally, the third model aims to test whether preoccupied workplace attachment style interacts with workplace bullying when predicting work engagement. Again, preoccupied workplace attachment style negatively impacts work engagement (LL = −0.30, UL = −0.10) and moderates the relationship between workplace bullying and work engagement (LL = 0.07, UL = 0.19), explaining 18% of the variance of work engagement (Table 7). The outcome pattern of preoccupied workplace attachment seems identical to that of avoidant workplace attachment. Preoccupied workplace attachment has a moderating effect at both low (*t* = −7.58, *p* < 0.001), mean (*t* = −7.20, *p* < 0.001), and high scores (*t* = −4.49, *p* < 0.001) (Table 8). The simple slope analysis (Figure 3) suggests that the lower their level of preoccupied workplace attachment, the more employees seem to disengage from their work in the face of workplace bullying (and vice-versa). This supports hypotheses 4 and 7.

**TABLE 7:** Moderation analysis of preoccupied workplace attachment.

	Work engagement			
			**95% CI**	
	**β**	**SE**	**LL**	**UL**
Workplace bullying	−0.36	0.05	−0.46	−0.26
Preoccupied workplace attachment	−0.20	0.05	−0.30	−0.10
Interaction	0.13	0.03	0.07	0.19
Covariates				
Age	0.07	0.04	−0.02	0.15
Sex	0.04	0.04	−0.05	0.12
	*R*^2^ = 18%			
	*F*_(5,466)_ = 20.77, *p* < 0.001			

**TABLE 8:** Conditional effect of focal predictor.

			95% CI	
**Preoccupied workplace attachment**	**Effect**	**SE**	**LL**	**UL**
−1SD	−0.49	0.06	−0.62	−0.36
Mean	−0.36	0.05	−0.46	−0.26
+1SD	−0.23	0.05	−0.33	−0.13

## 5. Discussion

The aim of this study was threefold. First, we questioned the well-established relationship between workplace bullying and work engagement using the Job Demands-Resources model (JD-R, Demerouti et al., 2001). We then examined the influence of secure and insecure (i.e., avoidant and preoccupied) workplace attachment styles on work engagement. Finally, we investigated how they interact with workplace bullying to predict work engagement in employees, expecting reverse buffering effects–that is, for dimensions associated with lower work engagement to have a protective effect on the relationship between bullying and work engagement (and vice-versa).

As expected regarding the main effect between workplace bullying and work engagement (*H*_1_), our results suggest employees who perceive being bullied are less engaged at work. This result echoes numerous past findings (e.g., Coetzee and van Dyk, 2018; Einarsen et al., 2018; Goodboy et al., 2020). According to the JD-R model, hindrance stressors such as workplace bullying participate in the impairment of employees’ wellbeing by causing various forms of strain and/or buffering the positive effect of job resources. As it does so *frequently* and *intensely* (Nielsen and Einarsen, 2018), workplace bullying may simultaneously discourage proactive behavior and foster a self-undermining process, both of which are associated with lower work engagement [see Bakker et al. (2023)]; the perspective of this vicious circle [and the associated *loss spiral* of resources, see Hobfoll et al. (2018)] may help clarify how targets of bullying are progressively rendered submissive to further abuse by perpetrators.

The following set of hypotheses claimed that a secure workplace attachment style would be positively related to work engagement (*H*_2_), whereas both avoidant (*H*_3_) and preoccupied (*H*_4_) workplace attachment styles would be negatively so. All of them were supported by the data. As predicted, employees interacting with the workplace as a secure base tend to rely on this personal resource to meet their needs (Scannell et al., 2020) or feel less strain (e.g., Scrima et al., 2021), which may foster more work engagement. Conversely, insecure employees either categorize their workplace as a job demand rather than a job resource (i.e., avoidant) or experience greater strain due to the relationship being anxiety-driven (Scrima et al., 2017); this may also explain why they tend to perceive more aggression coming from their work environment in the first place (Table 2). Moreover, these results support our initial suggestions that the physical work environment and secure workplace attachment, respectively act as a job resource and a personal resource in the context of the JD-R model (Bakker et al., 2023).

Hypotheses regarding the reverse buffering effects of avoidant (*H*_6_) and preoccupied (*H*_7_) workplace attachment styles on the workplace bullying–work engagement relationship were supported by the data. That is, the higher employees’ levels of either insecure workplace attachment style were, the less perceptions of bullying disengaged them from their work. From a statistical standpoint, our results do not support this reverse buffering effect when considering secure workplace attachment (*H*_5_); however, the associated conditional effects of workplace bullying on work engagement (Table 4) showed that employees with high levels of secure workplace attachment appear more impacted than others nonetheless. These results may be explained through the lens of *situational (in)congruence*, that is, the contrast (or lack thereof) between the relative expectations individuals have of their work environment and the actual experience of being bullied.

Past work suggests individuals will experience more positive and less negative affect when there is congruence between a given situation and their personality or self-concepts (Pervin, 1993, cited by Nielsen et al., 2022, p. 3) and vice-versa in case of incongruence (e.g., Ilies et al., 2011). This implies that the negative effect of workplace aggression should be more salient for individuals carrying positive representations of themselves and their environment [see Nielsen et al. (2008)]. Hence, employees with a positive view of themselves and their work environment (i.e., secure workplace attachment) should have all the more trouble resolving the incongruence caused by instances of workplace bullying, which may carry several consequences such as reduced work engagement (e.g., Mikkelsen and Einarsen, 2002). Previous work indeed shows that in situations of high environmental risk, individuals with a secure place attachment tend to feel more distress than others (Stancu et al., 2020). In threatening situations, insecure forms of attachment may provide ways to maintain congruence by confirming employees’ negative representations of either themselves (i.e., preoccupied) or their environment (i.e., avoidant); employees with an insecure workplace attachment may thus benefit from a situational adaptive advantage to the particularly potent stressor that is workplace bullying [see Ein-Dor (2015)].

These processes can also be framed using the managerial literature on *cognitive dissonance* [see Hinojosa et al. (2017), for review]; perceiving aggression in the workplace may be considered a situation of dissonance arousal prompting specific cognitive inconsistencies depending on employees’ initial representations (i.e., workplace attachment styles). As unresolved cognitive discrepancies prevent effective action (Harmon-Jones et al., 2009), employees are motivated to reduce dissonance by adopting certain behaviors or adjusting their cognitions accordingly. When faced with bullying, avoidant employees may reduce the inconsistency between their attitude and their behavior by *physically* distancing themselves from their workplace. When targeted, preoccupied employees could decide to stay late at work so as to rationalize their continued commitment to a hostile environment. Finally, as it contrasts all the more with their positive representations, employees with a secure workplace attachment style may be required to produce greater efforts in order to reduce the dissonance caused by workplace bullying.

The results of this study must be taken with caution due to its limitations. First, the data was collected with a cross-sectional design. This method does not allow us to identify cause-and-effect relationships. However, we relied on scientific literature and logic to determine the hypothetical directions of the effects. Furthermore, the data were collected using self-report instruments often subject to the common method bias (Podsakoff et al., 2012). Although different measurement models were compared to exclude the presence of common method bias, these results should be confirmed using longitudinal or experimental designs. Future work may also clarify the extent to which workplace bullying perceptions could alter workplace attachment over time, for instance. Finally, it should be specified that the chosen measure of workplace bullying does not reflect objective bullying events but rather the employee’s perception of being bullied. Pietersen (2007) recommends the triangulation of data from multiple sources (i.e., self-reports and interviews) to go deeper into investigating experiences of bullying.

Despite these limitations, our results have theoretical and practical implications. From a theoretical perspective, our contribution is the first to empirically show that secure workplace attachment can be conceptualized as an individual predictor of work engagement (i.e., a *personal resource* in the JD-R model, Bakker et al., 2014, 2023). This implies a reciprocal relationship with related job resources (such as the physical aspects of the workplace) in facilitating employees’ work (Roskams and Haynes, 2021), which provides a new avenue for future research. Our findings also contribute to a growing literature (e.g., Ilies et al., 2011; Nielsen et al., 2017a,^2022^) suggesting many typically protective personal characteristics can only be considered so when employee exposure to workplace bullying is low. Using the versatility of the JD-R model, our work further integrates constructs originating from environmental psychology to the understanding of the pervasive organizational phenomenon that is workplace bullying.

Our findings also carry important practical implications. First, previous literature has acknowledged how managers may mobilize the physical aspects of the work environment to promote employee performance and innovation, for Vischer (2007), Oksanen and Ståhle (2013). Our results reinforce the view that, in order to encourage work engagement, management should consider the ability of the physical-spatial work environment to satisfy employees’ needs (such as privacy, Laurence et al., 2013) and facilitate goal achievement. Some research has shown that high levels of functional comfort (i.e., a workplace equipped with all the tools to perform tasks) are associated with high levels of work engagement (Feige et al., 2013). Related interventions could be applied both *a priori* (e.g., analyzing the various needs of workplace users, Moos, 1973) or *a posteriori* through a dynamic adaptation of physical elements of the workplace. For instance, reducing work density (Wêziak-Białowolska et al., 2018) or giving employees the opportunity to bring a personal sense of coherence to their workspace (Vogt et al., 2016) may be valid strategies to foster work engagement. Following the JD-R model, higher work engagement may also help team members internalize more secure working models of their work space (or alter insecure models) in a virtuous circle.

Finally, the uncovered reverse buffering effects regarding avoidant and preoccupied workplace attachment styles should not be interpreted as an encouragement to promote these forms of affective bonds. As past literature described numerous benefits to employees having a secure (workplace) attachment (e.g., Scrima et al., 2021; Bruny et al., 2022), our results rather highlight the importance of preventing workplace bullying from arising in the first place. Identifying the workplace attachment style of team members may also clarify which are especially vulnerable to the effects of workplace aggression and how to best reinvigorate them once it has subsided–that is, help practitioners determine what type of interventions may benefit them most based on the affective bond they share with their workplace. In conclusion, it seems desirable for managers to (1) identify and acknowledge employees’ workplace attachment, (2) develop conditions encouraging a secure affective bond to the workplace, and (3) ensure the proximity thus desired by employees does not foster disengagement by preventing the emergence of workplace bullying. We propose they may rely on a culture of both *trust* and *justice* to do so (St-Pierre and Holmes, 2010).

## Data availability statement

The raw data supporting the conclusions of this article will be made available by the authors, without undue reservation.

## Ethics statement

Ethical review and approval was not required for the study on human participants in accordance with the local legislation and institutional requirements. The study was conducted in accordance with the Declaration of Helsinki and the APA’s ethical principles and code of conduct with human participants. The participants provided their written informed consent to participate in this study.

## Author contributions

KR and FS: conceptualization and formal analysis. FS, PI, and J-FH: methodology. FS, J-FH, KR, and PI: writing—original draft preparation. FS and J-FH: writing—review and editing. FS: visualization. PI and FS: supervision. All authors contributed to the article and approved the submitted version.
